# Prognostic role of neutrophil-to-lymphocyte ratio and platelet-to-lymphocyte ratio in patients with bone metastases

**DOI:** 10.1038/s41416-018-0231-6

**Published:** 2018-08-17

**Authors:** Quirina C. B. S. Thio, W. Alexander Goudriaan, Stein J. Janssen, Nuno Rui Paulino Pereira, Daniel M. Sciubba, Rachel P. Rosovksy, Joseph H. Schwab

**Affiliations:** 1 Department of Orthopaedics, Division of Orthopaedic Oncology, Massachusetts General Hospital – Harvard Medical School, 55 Fruit Street, Boston, MA 02114 USA; 2 Department of Orthopaedic Surgery, Academic Medical Center Amsterdam, Meibergdreef 9, 1105AZ Amsterdam-Zuidoost, Netherlands; 3 Department of Neurosurgery, The Johns Hopkins Hospital – The John Hopkins University School of Medicine, 1800 Orleans Street, Baltimore, MD 21287 USA; 4 Department of Medicine, Division of Hematology/Oncology, Massachusetts General Hospital – Harvard Medical School, 55 Fruit Street, Boston, MA 02114 USA

**Keywords:** Prognostic markers, Surgical oncology

## Abstract

**Background:**

Skeletal metastases are a common problem in patients with cancer, and surgical decision making depends on multiple factors including life expectancy. Identification of new prognostic factors can improve survival estimation and guide healthcare providers in surgical decision making. In this study, we aim to determine the prognostic value of neutrophil/lymphocyte ratio (NLR) and platelet/lymphocyte ratio (PLR) in patients with bone metastasis.

**Methods:**

One thousand and twelve patients from two tertiary referral centers between 2002 and 2014 met the inclusion criteria. Bivariate and multivariate Cox regression analyses were performed to determine the association of NLR and PLR with survival.

**Results:**

At 3 months, 84.0% of the patients with low NLR were alive versus 61.3% of the patients with a high NLR (*p* < 0.001), and 75.8% of the patients with a low PLR were alive versus 55.6% of the patients with a high PLR (*p* < 0.001). Both elevated NLR and elevated PLR were independently associated with worse survival (hazard ratio (HR): 1.311; 95% confidence interval (CI): 1.117–1.538; *p* = 0.001) and (HR: 1.358; 95% CI: 1.152–1.601; *p* < 0.001), respectively.

**Conclusion:**

This study showed both NLR and PLR to be independently associated with survival in patients who were treated for skeletal metastasis.

## Introduction

Bone is the third most common site of cancer metastasis, after lung and liver.^1^ Patients with malignant neoplasms such as lung, breast, renal, thyroid, and prostate carcinoma are especially prone to developing skeletal metastases.^2^ In the United States, the prevalence of adults with metastatic bone disease has been estimated to be over 280,000.^3^ When cancer metastasizes to bone, it can dramatically affect a patient’s quality of life. Patients may experience bone pain and—especially when the affected bone is load bearing—they can suffer pathological fractures.^2^ Pathologic fractures have been associated with worse quality of life and increased anxiety in addition to worsened pain.^4^ For that reason, surgery is often considered to prevent a pathologic fracture as well as to treat a pathologic fracture.

However, surgery itself is associated with morbidity. In patients, whose life expectancy is relatively short, one must balance the benefits of surgery versus the morbidity caused by surgery. In order to fully evaluate the potential benefits of surgery one must have an understanding of the patient’s life expectancy.^1,5,6^ Several prognostication tools have been developed to assist surgeons in predicting patient’s life expectancy.^6–9^ While these tools are useful, there is room for improvement. In order to improve the accuracy of these prognostication tools, it is important to consider new factors as they become available.

One of the hallmarks of cancer is inflammation and components of the inflammatory response, such as pro-inflammatory cytokines and chemokines, are often present in the tumor microenvironment.^10–12^ Two of these components, the neutrophil/lymphocyte ratio (NLR) and the platelet/lymphocyte ratio (PLR), have been described in two recent systematic reviews as prognostic indicators in various cancer types.^13,14^ The general thought is that inflammation stimulates the production and release of neutrophils and simultaneously decreases the production of lymphocytes.^15^ An increase in NLR can mean an increase in neutrophils, decrease in lymphocytes, or both. NLR and PLR can be measured in peripheral blood and may provide an easy and cost-effective biomarker for survival in patients with bone metastasis. For this specific group of patients, the prognostic value of NLR and PLR is not entirely clear. A previous study has investigated NLR in patients with skeletal metastases, of which half were surgically treated, and found it to be a prognostic factor.^16^ In this study, we aim to confirm their findings in a bigger cohort and additionally determine whether PLR is independently associated with worse overall survival in patients with skeletal metastasis.

## Materials and methods

### Data collection

This is a retrospective study, approved by our institutional review board with a waiver of consent. Data for this study were obtained from two tertiary care centers between January 2002 and January 2014. All patients older than 18 years of age that were surgically treated for a long bone or spinal metastasis were identified by searching the International Classification of Diseases, 9th revision (ICD-9) codes for metastatic long bone fracture or fracture of vertebrae. In addition, we used a word-based query to search operative reports on surgical management of a metastatic bone lesion in the spine or long bones. Multiple myeloma and lymphoma patients were also included, as from a practical perspective they are similar to patients with skeletal metastasis in terms of surgical decision making and orthopedic management. Exclusions included (1) patients undergoing revision procedures, (2) patients with metastatic involvement of the acetabulum or pelvis requiring reconstruction, (3) operative treatments other than endoprosthetic reconstruction, plate-screw fixation, intra-medullary nailing, and dynamic hip screw for long bone fractures, and (4) kyphoplasty and vertebroplasty for spinal fractures (rarely done and only in the most palliative setting). We chose these exclusion criteria to ensure our cohort was representative for the most common (impending) long bone and spine fractures and the most commonly chosen treatment procedures. If patients received multiple procedures for bone metastases, only the first procedure was included so as to not violate the statistical assumption of independence.

Only patients with pre-operative complete blood counts were included in this study. Of the 1295 consecutive patients, 1012 (78%) were included with available data for pre-treatment differential blood counts. Typically, all patients get pre-operative work-up with laboratory values. However, patients that were transferred from elsewhere may have had their lab results on paper which has not been added to their electronic medical chart.

### Outcome variables

The primary outcome was survival. The date of death was obtained from medical records and the Social Security Death Index, which is a database of death records created from the US Social Security Administration.^17^ The medical records and Social Security Death Index were last checked 11 June 2018. At 3 months, 95.9% of the patients who were not reported as deceased were still in active follow-up, at 6 months 93.5%, at 1 year 88.6%, and at 2 years 76.4%.

### Explanatory variables

Complete blood counts were extracted from the patient’s hospital record and the values closest to the date of surgery, but not more than 1 week preoperatively were included. The NLR was defined as the absolute neutrophil count divided by the absolute lymphocyte count, and the PLR was defined as the absolute platelet count divided by the absolute lymphocyte count. Primary tumor types were identified and categorized into three groups based on a study by Katagiri et al.^7^ and included slow growth (multiple myeloma, malignant lymphoma, thyroid cancer, hormone-dependent prostate cancer, and hormone-dependent breast cancer), moderate growth (lung cancer treated with molecularly targeted drugs, hormone-independent breast and prostate cancer, renal cell carcinoma, endometrial and ovarian cancer, sarcoma, and other), and rapid growth (lung cancer without molecularly targeted drugs, colorectal cancer, gastric cancer, pancreatic cancer, head and neck cancer, esophageal cancer, other urological cancers, melanoma, hepatocellular carcinoma, gall bladder cancer, cervical cancer, and cancers of unknown origin). Skeletal location was dichotomized into extremity and spine. Other metastases (in addition to the affected bone) were categorized as those with or without other bone metastases and with or without visceral (lung/liver/brain) metastases. Comorbidity was scored according to the Charlson Comorbidity Score and dichotomized in groups with or without additional comorbidity in addition to “metastatic tumor.” The location of the bones was dichotomized in spine and long bone. Previous radiotherapy of the affected bone was also noted as was the previous systemic therapy, which included chemotherapy, immunotherapy, hormone therapy, and metabolic therapy.

### Statistics

Baseline characteristics are displayed as frequencies and percentages for categorical variables, and median and interquartile range (IQR) for continuous variables.

When appropriate, the Fisher’s exact test was used to determine the strength and relationship between categorical variables. The optimal cutoff value of NLR and PLR was determined using the biostatistical tool Cutoff Finder by finding the most significant log rank.^18^ After determining these cutoff values, they were manually checked by performing log-rank tests with values closest to the determined value.

For the survival analyses, the Kaplan–Meier estimator with log-rank test for significance and Cox proportional hazard models were used for NLR and PLR. Patients that were lost to follow-up were censored at the time of last known contact. Additionally, cutoff values that were described in previous papers were used for sensitivity analyses. These values were 3, 4, and 5 for NLR^13,16^ and 150 and 300 for PLR.^14^ Bivariate Cox regression analyses were performed to determine which factors were significantly associated with survival. Variable body mass index (BMI) had missing cases and was analyzed using case-wise deletion. All variables with a *p* value <0.10 in bivariate testing, were tested in multivariable Cox regression to determine if NLR and PLR were independently associated with survival, and multiple imputations (40 imputations) were used to replace missing values for BMI.

All statistical tests were two sided and *p* < 0.05 was considered statistically significant. Statistical analyses were performed using StataCorp. 2013 (Stata Statistical Software: Release 13; StataCorp LP, College Station, TX, USA).

## Results

### Baseline characteristics

Baseline characteristics are shown in Table 1. The median age was 62 years (IQR 54–70) and sex was equally distributed. Of the bones affected, the femur was the most common location (*n* = 404), followed by the thoracic spine (*n* = 290), and the humerus (*n* = 113). The majority of the patients (66%) had multiple bone metastases (*n* = 666) and 43% of the patients had visceral metastases (*n* = 439). Most patients (61%) received previous systemic therapy (*n* = 614) and 26% received radiotherapy to the affected bone (*n* = 267). The median NLR and PLR before surgery were 6.4 (IQR 3.6–11.8) and 283 (IQR 174–452), respectively. The tumor distribution and primary tumor categorization, as described earlier,^7^ is shown in Table 2. Lung cancer (21%, *n* = 213) was the most common primary tumor, followed by breast cancer (17%, *n* = 177).

**Table 1:** Baseline characteristics

	*N*/median (%/IQR)^a^
Demographics	
Age (years)	62 (54–70)
Men	508 (50)
BMI (kg/m^2^)^b^	26.5 (23.2–29.8)
Race^c^	
White	790 (90.0)
Black	43 (4.9)
Asian	15 (1.7)
Other	30 (3.4)
Modified Charlson Comorbidity Index	6 (6.0–8.0)
Disease factors	
Pathologic fracture	399 (61)
Anatomic location	
Cervical spine	67 (6.6)
Thoracic spine	290 (28.7)
Lumbar spine	88 (8.7)
Combined spine	30 (3.0)
Femur	404 (40.0)
Humerus	113 (11.2)
Tibia	16 (1.6)
Radius	2 (0.2)
Ulna	2 (0.2)
Oncologic status	
Multiple bone metastases	666 (66)
Visceral/brain metastases	439 (43)
Previous systemic therapy	614 (61)
Previous local radiotherapy to the affected bone	267 (26)
Laboratory values^d,e^	
Hemoglobin (g/dL)^f^	11.1 (10.1–12.3)
Platelets (×10^3^/μL)	261 (194–342)
White blood cell count (/μL)	7900 (5700–10,500)
Neutrophils (/μL)	6100 (4100–8500)
Lymphocytes (/μL)	880 (560–1450)
Neutrophil-to-lymphocyte ratio	6.4 (3.56–11.84)
Platelet-to-lymphocyte ratio	283 (174–452)

^a^Due to rounding, percentages may not always appear to add up to 100%

^b^BMI was only available for 88.3% (894 of 1012) of the patients

^c^Race was missing in 13.2% (134 of 1012) of the patients

^d^Within a week before surgery

^e^SI conversion factors: to convert hemoglobin to g/L, multiply values by 10. To convert platelets to ×10^9^/L, multiply values by 1. To convert white blood cell count, neutrophils, and lymphocytes to ×10^9^/L, multiply values by 0.001

**Table 2:** Tumor distribution and categorization

Tumor distribution	*N*	%	Group
Lung cancer			
All except NSCLC with molecularly targeted therapy^a^	187	18	R
NSCLC with molecularly targeted therapy	26	2.6	M
Multiple myeloma	138	14	S
Breast cancer			
Hormone dependent	137	14	S
Hormone independent	40	4	M
Renal cell carcinoma	95	9.4	M
Malignant lymphoma	43	4.3	S
Malignant melanoma	42	4.2	R
Prostate cancer			
Hormone dependent	36	3.6	M
Hormone independent	36	3.6	S
Head and neck cancer	28	2.8	R
Colon and rectal cancer	27	2.7	R
NSCLC with molecularly targeted therapy	26	2.6	M
Esophageal cancer	25	2.5	R
Sarcoma	25	2.5	M
Thyroid cancer	20	2	S
Hepatocellular carcinoma	16	1.6	R
Other gynecological cancer	14	1.4	M
Other urological cancer	11	1.1	R
Pancreatic cancer	6	0.6	R
Gallbladder cancer	2	0.2	R
Gastric cancer	1	0.1	R
Cervical cancer	1	0.1	R
Unknown primary	18	1.8	R
Other^b^	38	3.8	M

*R* rapid growth, *M* moderate growth, *S* slow growth, *NSC* non small cell lung cancer

^a^Moleculary targeted agents: gefetinib and/or erlotinib

^b^Neuroendocrine (*n* = 21), testicular (*n* = 7), squamous cell carcinoma of the skin (*n* = 3), adrenal (*n* = 2), basal cell (*n* = 2), mesothelioma (*n* = 1), and paraglioma (*n* = 1)

### Survival

**Fig. 1:**
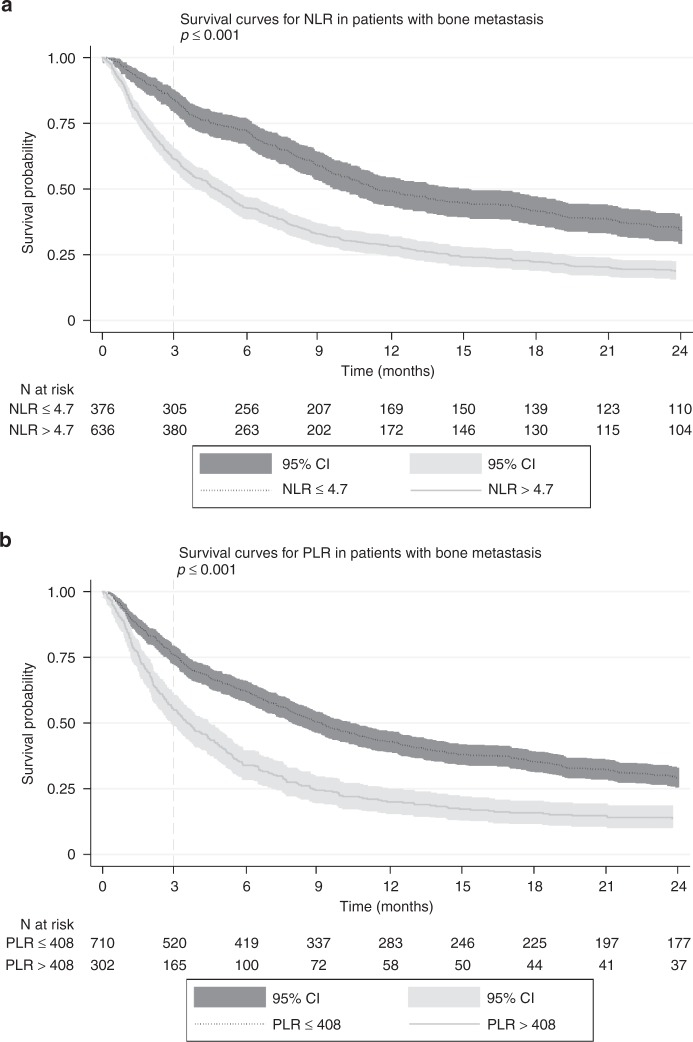
Kaplan–Meier survival curves for **a** NLR and **b** PLR in patients with bone metastasis. NLR neutrophil-to-lymphocyte ratio, PLR platelet-to-lymphocyte ratio

**Fig. 2:**
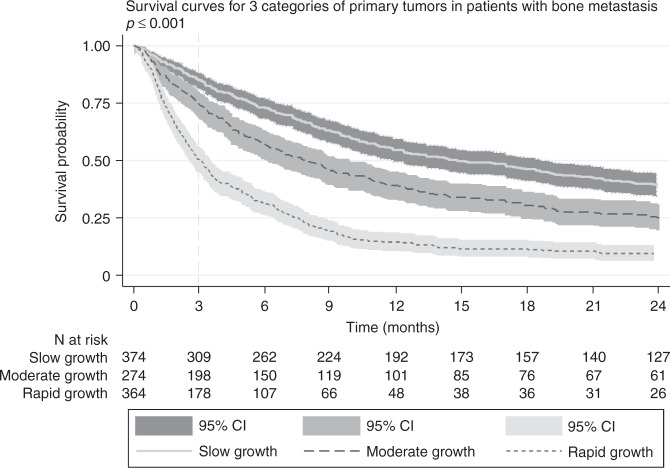
Primary tumors are categorized in slow growth, moderate growth, and rapid growth primary tumors

The median OS for all patients was 195 days (IQR 66.0–579.5). One month, 3 months, and 6 months survival were 91.0, 79.3, and 70.6%, respectively, and after 1 year the OS was 38.0%. Elevated NLR was associated with worse survival (Fig. 1a). At 3 months, 84.0% of the patients with an NLR under the cutoff value were alive versus 61.3% of the patients with an NLR above the cutoff value (*p* < 0.001). Elevated PLR was also associated with worse survival (Fig. 1b). At 3 months, 75.8% of the patients with a PLR under the cutoff value were alive versus 55.6% of the patients with a PLR above the cutoff value (*p* < 0.001). Figure 2 shows the Kaplan–Meier Survival curves for the three categories of primary tumors. There was a significant difference in overall survival between the three categories. Patients in the “rapid growth” category had the worse overall survival, and patients in the “slow growth” category had the best overall survival (*p* < 0.001). In bivariate analysis (Table 3), age (*p* = 0.005), BMI (*p* < 0.001), location of skeleton (*p* = 0.022), primary tumor category (*p* < 0.001), presence of other bone metastases (*p* = 0.002), presence of visceral metastases (*p* < 0.001), previous systemic therapy (*p* < 0.001), hemoglobin (*p* < 0.001), NLR (*p* < 0.001), and PLR (*p* < 0.001) were significantly associated with survival. Bivariate sensitivity analyses with cutoff values from previous literature for NLR and PLR were also significant. In the multivariate analysis, both NLR and PLR remained significant (Table 4).

**Table 3:** Bivariate Cox regression analysis

	HR	95% Confidence interval	*p* value
Age	1.008	1.002–1.014	0.005
Gender	0.970	0.848–1.109	0.654
BMI	0.968	0.955–0.982	<0.001
Race			
White	Reference		
Black	0.903	0.634–1.287	0.573
Asian	1.533	0.885–2.656	0.128
Other	0.867	0.562–1.339	0.521
Additional comorbidities	1.134	0.992–1.298	0.066
Location skeleton	1.171	1.023–1.340	0.022
Primary tumor			
Category 1 (slow growth)	Reference		
Category 2 (moderate growth)	1.566	1.316–1.863	<0.001
Category 3 (rapid growth)	3.071	2.603–3.624	<0.001
Other bone metastases	1.255	1.088–1.449	0.002
Visceral metastases	1.975	1.723–2.266	<0.001
Previous local radiation	1.121	0.965–1.303	0.136
Previous systemic therapy	1.508	1.310–1.736	<0.001
Hemoglobin	0.875	0.839–0.913	<0.001
**NLR** **(4.7)**	1.594	1.384–1.836	<0.001
NLR (5.0)	1.558	1.356–1.791	<0.001
NLR (4.0)	1.545	1.329–1.795	<0.001
NLR (3.0)	1.579	1.320–1.890	<0.001
**PLR** **(408)**	1.747	1.512–2.018	<0.001
PLR (150)	1.339	1.120–1.601	0.001
PLR (300)	1.391	1.215–1.592	<0.001

*NLR* neutrophil-to-lymphocyte ratio, *PLR*  platelet-to-lymphocyte ratio

**Table 4:** Hazard ratios for survival from stepwise, backward, and multivariate Cox hazard regression after multiple imputation (40 imputations)

	HR	95% Confidence interval	*p* value
Age	1.013	1.007–0.019	<0.001
Location skeleton	0.167	1.010–1.349	0.036
Category 1 (slow growth)	Reference		
Category 2 (moderate growth)	1.580	1.318–1.895	<0.001
Category 3 (rapid growth)	2.918	2.450–3.475	<0.001
Other bone metastases	1.201	1.029–1.401	0.020
Visceral metastases	1.544	1.333–1.789	<0.001
Previous systemic therapy	1.233	1.063–1.431	0.006
Hemoglobin	0.890	0.851–0.931	<0.001
**NLR**	**1.311**	**1.117–1.538**	**0.001**
**PLR**	**1.358**	**1.152–1.601**	**<0.001**

*NLR* neutrophil-to-lymphocyte ratio, *PLR*  platelet-to-lymphocyte ratio

## Discussion

In this study, a higher pre-treatment NLR and PLR were both significantly and independently associated with worse survival in patients who were surgically treated for skeletal metastasis of the spine or long bone.

The mechanisms behind the association of a high NLR and poor survival in patients with cancer are still poorly understood. In the nineteenth century links between inflammation and cancer were first suggested, as tumors were often found to arise from sites of chronic inflammation.^19^ A century later, leukocytosis in non-hematological cancers was found to be associated with poorer survival which was mainly due to an increase of neutrophils.^10,20^ In some cancer types, inflammatory conditions exist before malignant transformation, while in other types tumor development induces inflammation which leads to further tumor proliferation and growth.^11,12^ While tumors can produce cytokines and chemokines that become systemic, the tumor microenvironment is compartmentalized and the circulation does not directly reflect the tumor microenvironment.^21^ Furthermore, the role of neutrophils remains a controversial subject. On the one hand, neutrophils play a crucial role in the immune response, for instance, by recognizing and killing invading microorganisms through cytotoxic mechanisms.^22^ On the other hand, they are thought to inhibit the immune response by suppressing cytolytic immune cells, such as lymphocytes, and additionally to promote tumor growth by releasing tumor growth promoting factors like vascular endothelial growth factor in patients with cancer.^23–25^ Tumor-infiltrating lymphocytes have been shown to correlate with a good prognosis, for instance, due to the production of the cytokines interleukin-2 and interferon-γ.^26,27^ However, it is unclear to which extent levels of lymphocytes in the tumor microenvironment and the circulation correspond.

Although the complex mechanisms between cancer and circulating inflammatory components still remain to be unraveled, numerous studies have shown that elevated NLR is associated with adverse survival in various cancers, such as gastric, colorectal, lung, breast, endometrial cancer and multiple myeloma.^28–33^ A meta-analysis of 40,559 patients with solid malignancies found that an elevated NLR greater than the median cutoff of 4 was associated with worse OS (hazard ratio (HR) 1.81, 95% confidence interval (CI): 1.67–1.97).^13^ Wang et al.^16^ aimed to determine the prognostic implications of NLR inpatients with bone metastasis. They randomly selected 497 patients who were diagnosed with bone metastasis from different types of carcinoma, of which 225 were surgically treated. The method they used to determine the cutoff for NLR is unclear, but they dichotomized NLR in groups of ≤3.0 and >3.0. The authors found that a high NLR was associated with poor prognosis in the whole cohort (HR 1.348; 95% CI: 1.062–1.712, *p* = 0.014) and in the surgery group specifically (HR 2.945; 95% CI: 1.256–6.906, *p* = 0.013). In our study, we validate their findings.

One of the strengths of our study is the size of our cohort and the number of factors we considered, including the category of primary tumor. Furthermore, we used different cutoff values to evaluate the prognostic value of NLR and PLR. We chose to dichotomize our cutoff values by using the program Cutoff Finder which is a validated statistical method to determine a cutoff value, and additionally we did sensitivity analyses with values used in previous literature. Using Cutoff Finder, our optimal cutoff value for NLR was comparable to other studies. The meta-analysis by Templeton et al.^13^ showed that the cutoff values ranged from 1.9 to 7.2 with a median of 4.

The value of PLR as prognostic factor has also been studied extensively in the past decade. Several studies have shown that PLR is associated with poorer survival in patients with cancers such as ovarian, endometrial, gastric, breast, hepatocellular cancer, and cholangiocarcinoma.^31,32,34–37^ The mechanisms behind the association of high PLR and poor cancer prognosis remain unclear. Platelets may contribute to malignant progression by producing inflammatory cytokines and chemokines and by promoting tumor angiogenesis.^19^ A meta-analysis of 12,754 patients from 20 studies on PLR in solid tumors showed a significant association of higher PLR with worse OS and found that the size of effect of PLR on OS was greater for metastatic disease than early-stage disease.^14^

Even though there are many studies on the prognostic value of PLR in patients with specific types of cancer, there are no studies that have shown PLR to be an independent risk factor in this patient population. In our cohort, we found PLR to be an independent prognostic factor. The optimal cutoff value for PLR was 408, which is higher than that described in previous studies. The studies included in the meta-analysis of Templeton et al.^13^ which included patients with different types of cancers and in different stages of the disease, had cutoff values for PLR ranging from 150 to 300. The median platelet level in our study was 261 with an IQR of 194–342, which is higher than normal. The advanced stage of the disease which means a higher level of inflammation might mean a higher PLR. This in turn would mean that the cutoff point to make a distinction between prognosis may be higher too. The cutoff value we determined may therefore be more accurate for this patient population with advanced disease. We performed sensitivity analyses with 150 and 300 and PLR remained significant.

The use of different cutoff values for both NLR and PLR has been a subject of debate.^15,38^ A systematic review carried out by Dolan et al.^39^ pointed out that there are differences in mean ratios between different races. In this present study, race was not associated with survival, and after adding race to the multivariate analysis, both ratios remained significant. To prevent loss of data, Vano et al.^15^ have suggested that NLR should be investigated as a continuous factor. However, we believe that dichotomization makes the ratios easier to interpret in the clinical setting. Future studies with an external cohort are needed to validate our findings and to further explore the proposed cutoff values for this group of patients.

In the current study, we included all primary tumor types. The prognostic value of NLR and PLR and the respective optimal cutoff values may vary between different tumor types,^13,14^ which may mean that separate tumor-specific analyses of NLR and PLR are necessary. However, from a practical perspective it is reasonable to consolidate the different tumor types for both ratios. After categorizing the primary tumor types into three categories, both NLR and PLR remained significant in the multivariate analysis. This suggests that the same cutoff values can be used in the clinical setting for all tumor types. Additionally, we performed the same analyses excluding the hematological tumors and the outcomes remained the same.

Lower hemoglobin levels have been reported to have an adverse influence on survival in patients with cancer, regardless of the primary tumor type.^40^ It has therefore previously been incorporated as important factor in several prognostication models for patients with skeletal metastases.^9,40,41^ We confirmed these findings in the present study, underlining its importance as prognostic marker for survival. Data on treating anemia in cancer patients, for instance, by using erythropoietic proteins in order to increase survival remain inconclusive.^42^

We acknowledge that our study has several limitations. First, due to the retrospective nature of our study, there were no uniform criteria to determine treatment. Patients who did not receive surgical treatment were not included. Therefore, our results may only apply to patients who are being considered for surgical treatment. Second, we identified patients by ICD-9 codes and word-based searches, and we may have missed eligible patients. Third, we only included patients with available neutrophil, lymphocyte, and platelet counts, which were available in 78% of the patients. We compared the characteristics of our cohort to patients with missing values for all variables of interest. Patients without available blood counts were younger (*p* = 0.009) and had a higher BMI (*p* = 0.001). Other than that, there were no significant differences. Excluding these patients may have influenced our results. Fourth, we used a statistical tool to define the optimal cutoff value for both NLR and PLR. This is a data-driven approach opposed to a hypothesis-driven approach. However, we additionally used cutoff values that were previously described in literature and found similar results. Fifth, we acknowledge that confounding factors such as chemotherapy, antibiotics, steroids, smoking, and infection may influence neutrophil, lymphocyte and/or platelet counts. Nevertheless, as the exact mechanisms of the prognostic value of NLR and PLR are still unclear, we argue that the pre-treatment values reflect the patients’ immune response at that exact moment, and that it may not be important to know precisely which specific factors influenced them.

## Conclusions

In this study, we found that both NLR and PLR were associated with OS in patients with skeletal metastasis who underwent surgery. These inexpensive and accessible biomarkers may be useful when determining the best therapy for these patients and should be considered in prognostication models.

## **Data availability:**

The data that support the findings of this study are available from the corresponding author, Q.C.B.S.T., upon reasonable request.
